# Synthesis of Turbostratic Graphene Derived from Biomass Waste Using Long Pulse Joule Heating Technique

**DOI:** 10.3390/nano15060468

**Published:** 2025-03-20

**Authors:** Sukasem Watcharamaisakul, Nisa Janphuang, Warisara Chueangam, Kriettisak Srisom, Anuchit Rueangwittayanon, Ukit Rittihong, Sarayut Tunmee, Narong Chanlek, Peerapol Pornsetmetakul, Warodom Wirojsirasak, Nantida Watanarojanaporn, Kampon Ruethaivanich, Pattanaphong Janphuang

**Affiliations:** 1 School of Ceramic Engineering, Institute of Engineering, Suranaree University of Technology, Nakhon Ratchasima 30000, Thailand; sukasem@sut.ac.th (S.W.); nisa@slri.or.th (N.J.); 2 Synchrotron Light Research Institute, Nakhon Ratchasima 30000, Thailandkriettisak@slri.or.th (K.S.); anuchit@slri.or.th (A.R.); ukit@slri.or.th (U.R.); sarayut@slri.or.th (S.T.); narong@slri.or.th (N.C.); 3 Mitr Phol Innovation and Research Center, Phu Khiao, Chaiyaphum 36110, Thailandwarodomw@mitrphol.com (W.W.); nantidaw@mitrphol.com (N.W.); kamponr@mitrphol.com (K.R.)

**Keywords:** DC long pulse Joule heating (DC-LPJH), biomass, turbostratic graphene

## Abstract

This study addresses the challenge of the scalable, cost-effective synthesis of high-quality turbostratic graphene from low-cost carbon sources, including biomass waste such as sugarcane leaves, bagasse, corncobs, and palm bunches, using the Direct Current Long Pulse Joule Heating (DC-LPJH) technique. By optimizing the carbonization process and blending biomass-derived carbon with carbon black and turbostratic graphene, the gram-scale production of turbostratic graphene was achieved in just a few seconds. The synthesis process involved applying an 18 kJ electrical energy pulse for 1.5 s, resulting in temperatures of approximately 3000 K that facilitated the transformation of the carbon atoms into well-ordered turbostratic graphene. Structural and morphological characterization via Raman spectroscopy revealed low-intensity or absent D bands, with a high I_2D_/I_G_ ratio (~0.8–1.2), indicating monolayer turbostratic graphene formation. X-ray photoelectron spectroscopy (XPS) identified sp^2^-hybridized carbon and oxygenated functional groups, while NEXAFS spectroscopy confirmed the presence of graphitic features and both sp^2^ and sp^3^ bonding states. Energy consumption calculations for the DC-LPJH process demonstrated approximately 10 kJ per gram, demonstrating the potential for cost-effective production. This work presents an efficient approach for producing high-quality turbostratic graphene from low-cost carbon sources, with applications in enhancing the properties of composites, polymers, and building materials.

## 1. Introduction

Graphene, a groundbreaking material composed of a single layer of carbon atoms arranged in a two-dimensional honeycomb lattice, was first discovered in 2004 by Andre Geim et al. [1]. Its remarkable properties, including being 200 times stronger than steel by weight, highly flexible, and more conductive than copper, make it ideal for a wide range of advanced materials and applications. Additionally, graphene exhibits exceptional thermal conductivity (approximately 5000 W/m·K), allowing for efficient heat dissipation, while its near-transparency, absorbing only about 2.3% of visible light, also makes graphene suitable for transparent conductive films. With these extraordinary properties, graphene has the potential to be used as a composite material in diverse fields: in electronics for faster and more efficient devices; in energy storage for enhanced battery and capacitor performance; in structural reinforcement for the aerospace, automotive, and construction industries; in water filtration and desalination systems, due to its impermeability; and in medicine for targeted drug delivery, bio-imaging, and advanced medical devices [2,3,4,5,6,7,8,9,10,11,12,13,14,15,16].

Graphene and graphene-like material synthesis methods can be categorized into top-down and bottom-up approaches. Top-down methods include mechanical exfoliation, which involves peeling graphite layers using adhesive tape. This method can yield pristine graphene flakes, but suffers from low yield and inconsistent layer thickness, making it less viable for large-scale applications [17]. Liquid-phase exfoliation uses solvents and sonication to separate graphite into graphene layers, offering scalability, but yielding lower-quality graphene with potential defects [16,17,18,19,20,21]. Unzipping carbon nanotubes can also structure graphene nanoribbons by cutting nanotubes chemically or using plasma [16,18]. Bottom-up methods include chemical vapor deposition (CVD), which deposits carbon atoms from hydro-carbon gases onto metal substrates like copper or nickel under high temperatures. CVD is widely used to produce high-quality, single-layer graphene with excellent electrical conductivity, high crystallinity, and minimal defects, making it suitable for electronics and sensors. However, CVD requires high temperatures and specific substrates, limiting its scalability and increasing production costs [18,19,20,21]. Single-layer and few-layer graphene can be an epitaxial growth on SiC substrates via a thermal decomposition process. The SiC crystal is heated at temperatures above 1000 °C. As a result, the Si sublimates from the surface in gaseous form, and the carbon atoms are left behind and rearrange to form a graphene layer. The main drawback to epitaxial growth is that it requires highly specialized equipment, as well as the cost of the single-crystal SiC substrate [16,18,19,20]. The arc discharge method employs high-temperature plasma generated by electric arcs to convert graphite into graphene, offering scalability but being energy-intensive [22,23,24]. Lastly, the reduction of graphene oxide (GO) involves oxidizing graphite into graphene oxide, followed by a chemical, thermal, or electrochemical reduction to graphene. This process is economical and scalable, but often introduces structural defects [16,17,19,21]. Table 1 summarizes the synthesis methods available for graphene and graphene-like structures.

Flash Joule Heating (FJH) is a recent advanced method for turbostratic graphene synthesis that offers significant advantages over traditional techniques like CVD or exfoliation. The FJH technique was first discovered and reported in 2020 [25]. This process rapidly heats carbon-containing materials to high temperatures (up to 3000 K) using a short high-electrical-energy pulse in an inert environment within milliseconds. The rapid heating causes the decomposition of the precursor material, breaking the non-carbon bonds and allowing the carbon atoms to rearrange into thin-sheet graphene-like structures. FJH stands out for its ability to use inexpensive or waste carbon sources [25,26,27,28,29,30,31]. It is highly scalable, suitable for industrial-scale production, and capable of producing large quantities of turbostratic graphene efficiently. FJH can typically produce “turbostratic” graphene, where misaligned layers reduce interlayer adhesion, simplifying its integration into composites [25]. Moreover, a more detailed description of the growth mechanism of graphene-like materials influenced by the interactions of carbon atoms, their bonding states, and their external synthesis conditions has been illustrated in [31]. From a theoretical perspective, the Synthetic Growth Concept (SGC) provides a framework for better understanding the interplay between the bonding, hybridization, and structural evolution of turbostratic graphene during FJH. This concept initially introduced the formation of graphene layers with varying degrees of order and guided the synthesis of disordered turbostratic graphene [32,33,34].

**Table 1:** Comparison of synthesis methods for graphene and graphene-like materials.

	Flash Joule Heating (FJH) [25,27,29]	Chemical Vapor Deposition (CVD) [18,19,20,21]	Mechanical Exfoliation [16,17,18,19,20,21]	Liquid-Phase Exfoliation [16,17,18,19,20,21]	Epitaxial Growth on SiC [16,18,19,20]
Synthesis Time	Milliseconds	Hours	Time-intensive (manual process)	Hours to days	Hours to days
Temperature	2000–3000 K	~1000–1300 K	Room temperature	Room temperature or mild heating	>1000 °C
Scalability	High (bulk synthesis)	Limited (substrate-dependent)	Very low (small flakes)	Moderate (bulk possible)	Limited (substrate-dependent)
Purity	High (minimal oxygen, few defects)	High (single/few layers)	High (monolayer possible)	Moderate (polycrystalline structure)	High (epitaxial layers)
Defect Density	Low (turbostratic graphene)	Low (depending on transfer)	Very low	High (defects from sonication)	Very low
Cost	Low (cheap carbon feedstocks)	High (substrates, gases)	Low (but inefficient)	Low to moderate (depends on solvents, sonication)	Very high (SiC substrates)
Environmental Impact	Lower (no toxic chemicals)	Higher (methane, hydrogen)	Low	Moderate (solvent use)	High (energy-intensive process)
Conductivity	High (~10⁴ S/m)	Very high (~10^5^ S/m)	Very high	Moderate (~10^2^ to 10^3^ S/m)	Very high
Structural Order	Turbostratic graphene (loosely stacked layers, easy to exfoliate)	Crystalline (high-quality monolayers)	Crystalline monolayers (small flakes)	Polycrystalline sheets	Crystalline monolayers
Layer Control	Poor (randomly stacked layers)	Excellent (monolayer possible)	Excellent but hard to scale	Poor (varied thickness)	Excellent
Applications	Composites, coatings, energy storage, conductive inks	Electronics, transparent electrodes	Fundamental research, sensors	Composites, inks, coatings	High-speed electronics, quantum devices

Thailand generates a significant amount of biomass waste, primarily from agricultural activities. The extensive agricultural sector contributes to the production of various types of biomass waste, including rice husks, sugarcane bagasse, cassava residues, and palm oil residues. Most biomass waste is commonly disposed of through several methods, such as open burning, landfilling, or incineration. Among these, open burning is widely used due to its simplicity and low cost. However, this method poses significant environmental challenges, as it releases large amounts of pollutants, including greenhouse gases (e.g., CO_2_ and CH_4_), particulate matter, and toxic compounds. These emissions contribute to air pollution, global warming, and health hazards for nearby communities.

Recently, biomass waste has attracted significant interest as a new carbon source due to its carbon-rich structure and renewability. Biomass is primarily composed of cellulose, making it a promising material for graphene production, as cellulose can experience pseudomorphic transformation into porous carbon through carbonization [35]. Using biomass as a precursor for graphene production not only offers an opportunity to recycle agricultural waste but also provides a “carbon-neutral” solution, as it does not contribute to a net rise of carbon dioxide in the atmosphere. Furthermore, the natural permeability of the graphene-like structures derived from biomass makes them attractive candidates for various applications. In composite materials, incorporating turbostratic graphene can significantly enhance the mechanical properties, such as tensile strength and flexibility, due to its unique layered structure and strong interfacial interactions with the polymer matrix. Additionally, the electrical conductivity of turbostratic graphene can be leveraged to create conductive composites for use in electronic devices, sensors, and energy storage systems. Photoelectrochemical technologies, such as solar cells and water-splitting systems, stand out among these applications [36].

In this work, turbostratic graphene synthesized from biomass waste using the Direct Current Long Pulse Joule Heating (DC-LPJH) process has been demonstrated. The process started by converting biomass waste into carbon precursors using a carbonization process under an argon gas atmosphere at temperatures ranging from 400 °C to 800 °C. The carbon precursor powder was then initially stimulated by a high-electrical-energy pulse for a short period of 1.5 s, until it reached temperatures of about 3000 K (2727 °C), causing the carbon atoms to rearrange themselves into graphene-like structures. The turbostratic graphene obtained from the DC-LPJH process was analyzed and characterized through several spectroscopic techniques. Raman spectroscopy was used to confirm the structural integrity and defect density through the analysis of the D, G, and 2D bands. Transmission Electron Microscopy (TEM) provided insights into the morphology and graphene-like layer structure. In addition, X-ray photoelectron spectroscopy (XPS) revealed the elemental composition and bonding states, while synchrotron-based Near-Edge X-Ray Absorption Fine Structure (NEXAFS) spectroscopy was further used to verify the electronic structure and chemical bonding, indicating the successful synthesis of the turbostratic graphene derived from biomass waste.

## 2. Materials and Methods

### 2.1. Carbon Powder Preparation from Biomass Waste

The carbon precursor powder in this work was derived from various biomass waste sources, including bagasse, cassava pomace, sugarcane leaves, straw, palm bunches, corncobs, and corn stalks. The biomass waste was first mechanically milled to transform it into small particles in order to control the homogeneity and optimize the production yield of the carbonized materials. Moreover, the milling process significantly increases the surface area of the biomass-based particles, exposing more reactive sites for thermal decomposition. This enhances the carbonization efficiency and improves the quality of the carbon precursor for graphene production. Furthermore, a uniform particle size ensures consistent heating, reducing the risk of incomplete or uneven carbonization [37,38].

**Figure 1:**
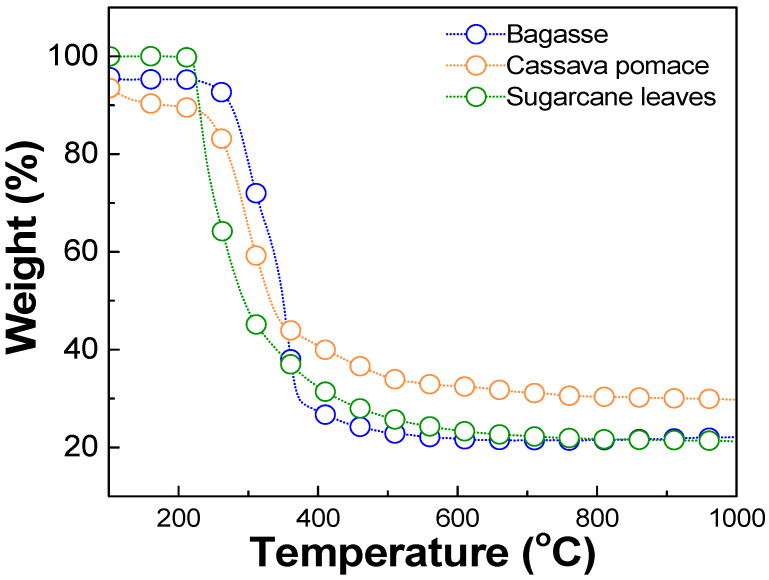
Thermogravimetric analysis (TGA) results of biomass waste, including bagasse, cassava pomace, and sugarcane leaves.

**Figure 2:**
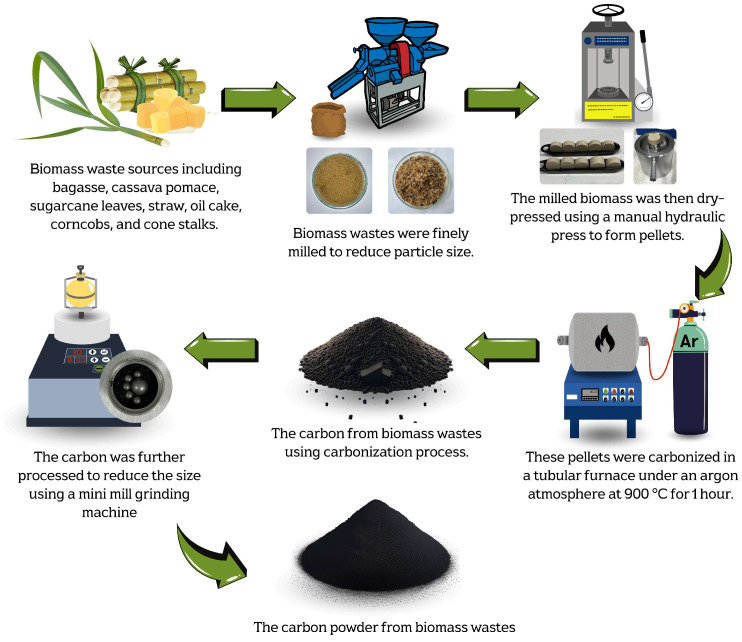
Schematic diagrams of carbonization process used to produce carbon powder from biomass waste.

The optimal carbonization temperature and the weight change of each biomass waste were determined using thermogravimetric analysis (TGA), DSC 214 Polyma, NETZSCH, Selb, Germany, TGA mode, as shown in Figure 1. The results revealed that each biomass exhibited significant weight loss within the temperature range of 220–250 °C, corresponding to the thermal decomposition of volatile compounds such as hemicellulose, cellulose, and other organic constituents [37,38,39]. Beyond 400 °C, the weight loss stabilized, and the residual weight remained relatively constant up to 1000 °C. This stable region indicated the completion of the carbonization. Therefore, a temperature in the range of 400–1000 °C was considered optimal for the carbonization process in this work. The milled biomass was then dry-pressed using a manual hydraulic press, applying a pressure of approximately 1.5–2 N, to form pellets. These pellets were carbonized in a tube furnace (FURNACE DESIGN, Model: FD-TUBE FURNACE, Lampang, Thailand) under an argon atmosphere with a flow rate of 100 sccm and at temperatures of about 900 °C for 1 h (ramp rate of 10 °C/min). A schematic diagram of the carbonization process is shown in Figure 2. Argon was used during the carbonization process, preventing oxidation and unwanted side reactions. This allowed for the gradual decomposition of volatile compounds, ensuring a high-purity carbon yield [35].

All biomass waste and carbonized carbon precursors used in this work were analyzed for their elemental composition using X-Ray Fluorescence (XRF) with a WD-XRF-instrument (ZSXPrimus IV, Rigaku Corporation, Tokyo, Japan). This analysis identifies inorganic elements such as metals, minerals, and trace elements. While XRF does not directly detect carbon, hydrogen, or oxygen, it reveals ash-forming elements, which are crucial for understanding biomass properties and combustion behavior. The elemental composition of the biomass can influence the purity, electrical conductivity, and yield efficiency of the graphene production. Palm bunches exhibited the highest carbon content of 80.29% after carbonization, primarily due to the high proportion of lignocellulosic material in the palm bunches. In contrast, cassava pomace had the lowest carbon content of 40.04% after carbonization. As shown in Table 2, the main elements in the cassava pomace were Calcium (Ca), Silicon (Si), Potassium (K), and Magnesium (Mg), with 22.24% of the composition being Ca. The presence of Ca leads to a reduction in carbon content during carbonization. Furthermore, it is evident that the presence of Si and Ca in the biomass, especially in samples like the straw and cassava pomace, plays a significant role in the synthesis of turbostratic graphene via the DC-LPJH process. Non-conductive elements or insulators hinder the efficient transfer of electrical energy during the DC-LPJH process, which is crucial for achieving the rapid and uniform heating needed to transform the carbon materials into high-quality turbostratic graphene. In biomass, Si typically exists as silica (SiO_2_), a strong insulator, while Ca can also be found in forms such as CaCO_3_, CaO, and Ca_3_(PO_4_)_2_, which act as insulators rather than conductors [29,40,41]. Thus, elevated Si and Ca content must be considered when it comes to the process of transformation of biomass to turbostratic graphene using DC-LPJH.

### 2.2. Graphene Synthesis Using DC-LPJH

**Figure 3:**
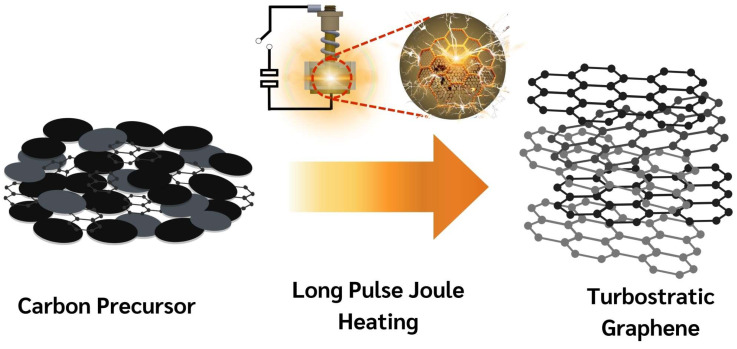
Schematic diagram of transformation mechanism of DC-LPJH synthesis.

**Figure 4:**
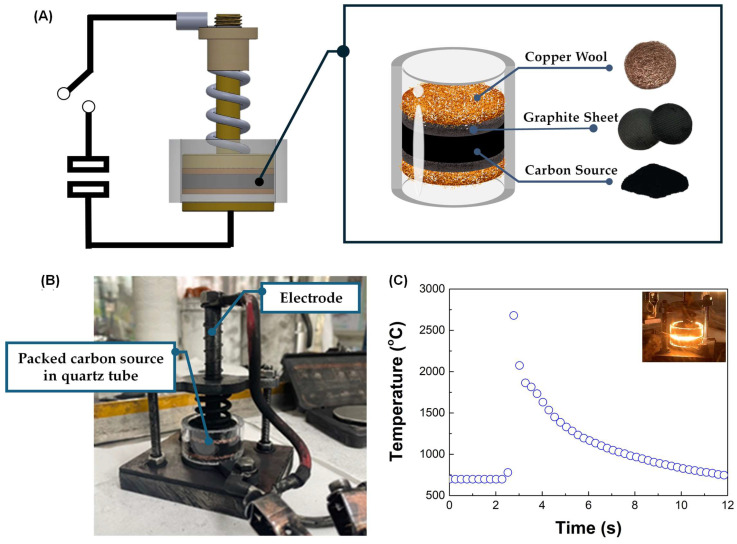
(**A**) The schematic diagram of the DC-LPJH turbostratic graphene synthesis. (**B**) The installation of the packed quartz tube in the vacuum chamber. (**C**) The temperature profile during the DC-LPJH process.

Flash Joule Heating (FJH) is a rapid and efficient method for synthesizing turbostratic graphene from carbon-based materials. The process involves applying a short pulse of high-voltage electric discharge, which rapidly heats the carbon source to temperatures exceeding 3000 K within milliseconds. This intense heat causes the decomposition of the precursor material, breaking the non-carbon bonds and enabling the carbon atoms to rearrange into thin-sheet graphene-like structures [25], as illustrated in Figure 3. The DC-LPJH developed in this work focused on the optimization of the energy efficiency and scalability of the synthesis to improve the structural quality and yield of the graphene-like structure derived from biomass waste. Since most biomass contain various elements, including carbon and insulators, as discussed in Section 2.1, these elements affect the conductivity of the carbon precursors. As a result, high energy stimulation over a longer period is necessary to ensure sufficient heating and facilitate the conversion of these materials into graphene-like structures [25]. To optimize the energy supply and improve the yield of the graphene production, this work utilized the addition of carbon black and the high electrical conductivity of turbostratic graphene to compensate for the conductivity of the carbon precursors derived from the biomass waste. The optimal weight ratio of the carbon precursors, carbon black (N220 ISAF, Continental Carbon^®^, Sunray, TX, USA) and turbostratic graphene was determined to be 50:30:20. A mixture of approximately 1.8 g of carbon powder, with an electrical resistivity ranging from 0.6 to 0.8 ohm depending on the type of biomass, was then loaded into a quartz tube sandwiched between two copper wool electrodes. The quartz tube had an inner diameter of 32 mm and a length of 23 mm, as illustrated in Figure 4.

A packed carbon-sourced tube was placed in a vacuum chamber and pumped down to a pressure of approximately 100 mTorr to reduce the oxygen and enhance the outgassing from the quartz tube (Figure 4B). The activation of the quartz tube was performed using DC-LPJH, delivering 14–18 kilojoules of electrical energy at 110 V over 1.5 s. During this process, a high-voltage discharge from a 6 mF capacitor bank passed through the electrodes into the mixed carbon, rapidly heating it to temperatures exceeding 3000 K, as measured by a pyrometer (Impac IS 140, Advanced Energy Industries, Inc., Fort Collins, CO, USA) (Figure 4C). The intense heat generated during the process broke the bonds between the carbon and gas molecules, allowing the carbon atoms to rearrange into graphene-like structures. The synthesized turbostratic graphene derived from the biomass waste was characterized using Raman spectroscopy (SENTERRA, Bruker Optics GmbH & Co. KG, Ettlingen, Germany) with a 532 nm diode laser for single-point measurements. The graphene-like layer structure and morphology were confirmed by Transmission Electron Microscopy (TEM) (TALOS F200X, Thermo Scientific, Waltham, MA, USA). To determine the purification level and confirm the removal of the inorganic contaminants, the synthesized turbostratic graphene from bagasse was selected for further characterization using synchrotron-based X-ray photoelectron spectroscopy (XPS) at Beamline 5.3, Synchrotron Light Research Institute (Public Organization), Nakhon Ratchasima, Thailand. Additionally, to analyze the atomic structure and purity of the turbostratic graphene, synchrotron-based Near-Edge X-Ray Absorption Fine Structure (NEXAFS) spectroscopy was conducted at Beamline 3.2, using photon energies ranging from 270 eV to 320 eV with the C K-edge.

## 3. Results

### 3.1. Characterization

#### 3.1.1. Raman SPECTROSCOPY

**Figure 5:**
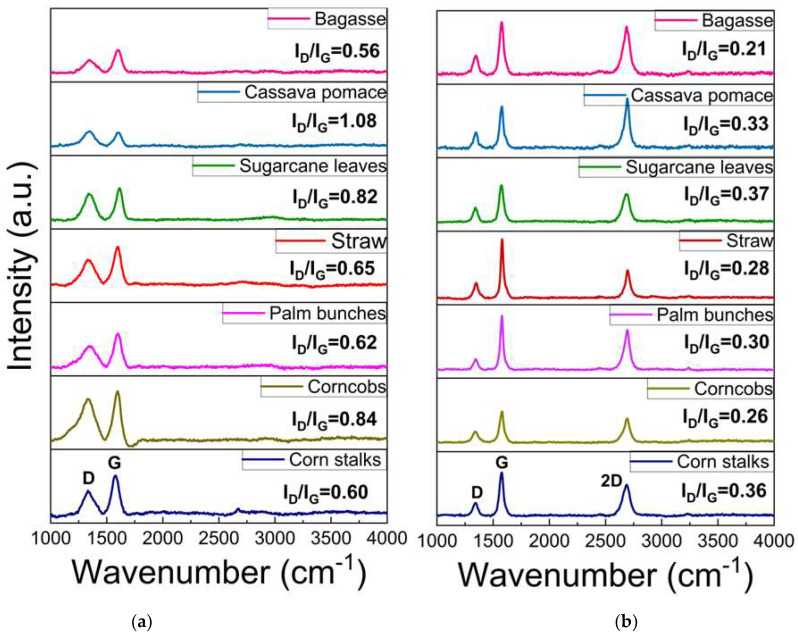
Raman spectroscopy of (**a**) carbon source and (**b**) turbostratic graphene from biomass.

**Figure 6:**
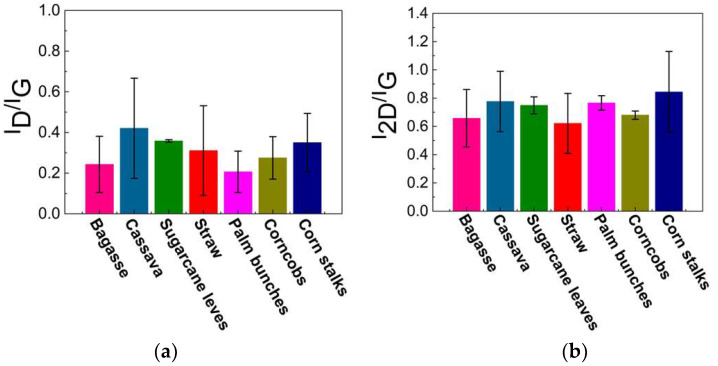
Calculated results of (**a**) I_D_/I_G_ and (**b**) I_2D_/I_G_ ratio.

Raman spectroscopy is a rapid technique used to identify high-quality graphene-like structures. The D band (around 1350 cm⁻^1^) is associated with structural defects or disorder in the carbon lattice, often indicating the presence of sp^3^ carbon or oxygen-containing functional groups. The G band (around 1590 cm⁻^1^) corresponds to the in-plane vibrations of sp^2^-hybridized carbon atoms exhibiting the characteristics of graphitic structures. This G band also provides information about the degree of order in the graphite. A peak around 2700 cm⁻^1^, known as the 2D band, is typically observed in graphitic or graphene-like structures, and indicates multi-layer stacking [40,41,42,43,44,45,46,47,48]. As illustrated in Figure 5a, the Raman spectrum of the carbon precursor derived from each biomass waste lacks a significant 2D band, indicating that the materials are not highly graphitized or graphene-like. In contrast, Figure 5b displays an intense 2D peak with a low-intensity D band, which suggests a low defect concentration and confirms the presence of turbostratic stacking in the graphene. The 2D peak position falls within the range of 2685–2700 cm⁻^1^, indicating the formation of turbostratic graphene. Slight variations among the different biomass sources suggest differences in the graphitization levels and structural order. Additionally, the I_D_/I_G_ ratio can be used to assess the defect density and structural disorder in the graphene-like materials. A higher I_D_/I_G_ ratio indicates structural defects, smaller graphene domains, or partially disordered carbon structures, while lower values suggest a more ordered graphene-like structure, as shown in Figure 6a. The I_2D_/I_G_ ratio (Figure 6b) provides further insights into the structural characteristics of the resulting material, particularly the degree of turbostratic disorder and layer stacking. Through all biomass-derived samples, the I_2D_/I_G_ ratio remains below 1.0, indicating the formation of few-layer turbostratic graphene rather than single-layer graphene. These moderate values suggest that the synthesized material exhibits loosely stacked layers with minimal interlayer interactions, a characteristic of turbostratic graphene. These variations highlight the influence of biomass composition on the structural properties of the synthesized graphene-like materials, emphasizing the importance of optimizing the precursor selection and processing conditions to achieve high-quality turbostratic graphene [46].

#### 3.1.2. Transmission Electron Microscopy (TEM)

**Figure 7:**
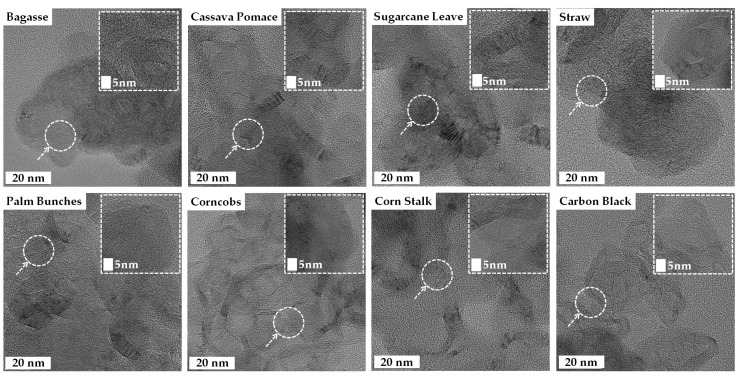
Transmission Electron Microscopy (TEM) images of turbostratic graphene derived from various biomass wastes.

The high-resolution TEM images of the turbostratic graphene reveal thin, sheet-like layers with some wrinkled structures, exhibiting varying degrees of crystalline and turbostratic disorder, as shown in Figure 7. The inset images at a 5 nm scale provide a closer look at the atomic lattice fringes, confirming the presence of graphitic domains. The observed interlayer spacing, likely around 0.33–0.36 nm, is characteristic of turbostratic graphene rather than well-ordered graphite [49]. The turbostratic graphene derived from the straw, sugarcane leaves, and bagasse displayed a more irregular, flaky texture, whereas the structures obtained from the corncobs, corn stalks, and palm bunches were more continuous and sheet-like [25,27,31,48,49,50]. The TEM results confirm the successful formation of graphene-like materials across all biomass sources.

#### 3.1.3. X-Ray Photoelectron Spectroscopy (XPS)

**Figure 8:**
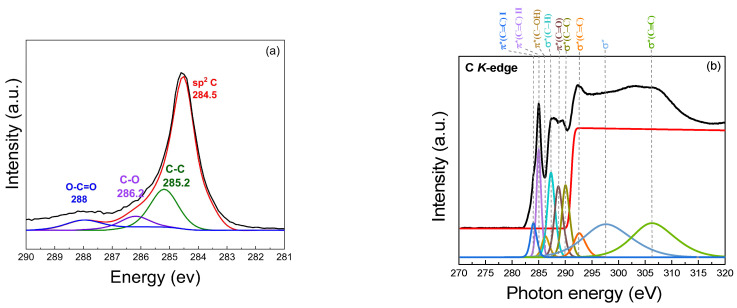
Chemical composition analysis: (**a**) XPS spectrum; (**b**) Near-Edge X-Ray Absorption Fine Structure (NEXAFS) of turbostratic graphene derived from bagasse.

The XPS spectrum provides insights into the chemical states presented in the samples, as illustrated in Figure 8a. A prominent binding energy peak at 284.5 eV (sp^2^ C) corresponds to sp^2^-hybridized carbon (C=C) bonds, demonstrating the presence of a graphene-like structure with a high degree of graphitic carbon. The peak at 285.2 eV is associated with the C–C bonds in the graphene-like structure. It represents the carbon atoms in the graphene structure that remain unoxidized, indicating sp^2^-hybridized carbon atoms in the graphene structure. The presence of carbon–oxygen (C–O) bonds, which may arise from hydroxyl or epoxy groups on the turbostratic graphene surface, is observed at 286.2 eV, indicating partial oxidation. Carboxyl (O–C=O) groups at a binding energy of 288 eV contribute to the oxidized state of the graphene-like structure. The presence of these oxygenated functional groups (C–O, O–C=O) demonstrates that the synthesized turbostratic graphene has experienced some degree of oxidation, which can influence its electronic and chemical properties, such as hydrophilicity [51,52,53,54,55,56]. The statistical analysis reveals that the mean peak area for the C1s component is 86.72%, with a standard deviation of 4.21%, while the O1s component exhibits a mean peak area of 13.24%, with a standard deviation of 4.26%. The presence of oxygen can be attributed to the exposure to air, leading to surface oxidation. Additionally, the calculated O/C ratio of 0.153 suggests a moderate level of oxygen functionalization, which is consistent with the characteristics of turbostratic graphene [57].

#### 3.1.4. Near-Edge X-Ray Absorption Fine Structure, NEXAFS

The atomic structure of the biomass-derived turbostratic graphene was investigated using NEXAFS at the carbon K-edge, as shown in Figure 8b. The results provided detailed insights into the sp^2^ character, layer stacking, and residual functional groups present in the graphene-like sheets. At the carbon K-edge, prominent features characteristic of sp^2^-hybridized carbon are observed. The π resonance*, located at 284.5 eV, corresponds to transitions from the C 1s core level to the unoccupied π* states of sp^2^ carbon. The sharpness and intensity of this peak, compared to the graphite, confirm the dominance of the well-ordered graphene-like structures. In addition to the π* and σ* resonances, several secondary features were observed, indicating the presence of oxygen-containing functional groups, likely retained from the biomass precursors. The peak near 287.0 eV corresponds to the π*(C=O) transition, associated with carbonyl groups. Similarly, a weak feature around 288.4 eV suggests the presence of carboxyl (O–C=O) functionalities. The peak at 289.6 eV represents the σ* state, indicating C–C bonds with an sp^3^ orbital structure, which is characteristic of diamond-like structures. Additional peaks are observed at 286.1 eV for π* (C–OH), 287.1 eV for σ* (C–H), 288.3 eV for π* (C=O), 291.0 eV for σ*_1_ (C=C), 292.4 eV for σ*_2_ (C=C), 301.0 eV for σ* (C–C), and 307.0 eV for σ* (C≡C) [53,58,59,60].

## 4. Discussion

### 4.1. Graphene Formation and Quality

The results presented in this study demonstrate the successful synthesis of turbostratic graphene from various biomass waste sources using the DC-LPFJH technique. The Raman spectroscopy, TEM, XPS, and NEXAFS data provide strong confirmation of the high-quality graphene-like structures obtained and highlight the influence of biomass composition on the final product. The Raman spectra of the carbon precursors derived from biomass waste materials exhibit a lack of a distinct 2D peak, confirming the absence of highly ordered graphitic structures prior to the DC-LPFJH process. This observation aligns with the unprocessed nature of the raw biomass, which typically contains non-graphitic carbon and other heteroatoms that hinder graphitization. After the DC-LPFJH process, the Raman spectra reveal a prominent 2D band around 2685–2700 cm⁻^1^, indicating the formation of turbostratic graphene. This result is in agreement with previous studies on graphene-like materials synthesized from carbon precursors, which have reported a characteristic 2D peak at similar positions [43,44,45,53].

The low-intensity D band observed after DC-LPFJH suggests minimal structural defects and a relatively high degree of graphitic ordering, which is desirable for applications requiring high-quality graphene. The I_D_/I_G_ ratio, which quantifies the defect density, further supports the high quality of the synthesized graphene. The low I_D_/I_G_ ratio obtained in this study indicates a well-ordered structure with fewer defects, a critical factor for optimizing the electrical and mechanical properties of the graphene. However, the slight variation in the I_D_/I_G_ ratio across the different biomass sources suggests that the composition of the precursor material plays a role in the final graphene-like structure. Biomass waste with higher carbon content yields graphene with lower defect densities, highlighting the importance of selecting suitable raw materials to achieve the desired graphene characteristics. The I_2D_/I_G_ ratio is a key indicator of the number of graphene layers and the level of crystallinity. In this study, the I_2D_/I_G_ ratios ranged from 0.5 to 1.2, suggesting a mixture of few-layer and more disordered graphene structures. Higher ratios (closer to 1.2) correspond to more ordered, fewer-layer graphene, which is generally preferred for applications in energy storage due to its high surface area and conductivity. On the other hand, lower I_2D_/I_G_ ratios (around 0.5) indicate increased disorder and thicker graphene layers, which are typical of turbostratic graphene, but may not be ideal for high-performance applications. The variations in the I_2D_/I_G_ ratio across the different biomass sources point to the influence of the precursor composition on the final graphene structure, with some sources producing more crystalline materials than others.

### 4.2. Morphological Characteristics

The high-resolution Transmission Electron Microscopy (TEM) images provide valuable insights into the morphology of the synthesized turbostratic graphene, revealing structural differences based on the type of biomass waste used. Graphene derived from straw, sugarcane leaves, and bagasse exhibits an irregular, flaky texture, while materials sourced from corn cobs, corn stalks, and palm bunches display a more continuous, sheet-like structure. These variations are attributed to the elemental composition and nature of the precursor biomass. Biomass with higher carbon content and lower levels of insulating elements, such as silicon and calcium, tends to produce more ordered graphene structures, indicating that the heteroatom content significantly influences the morphology and quality of the final product. The wrinkled, sheet-like nature observed in most samples is typical of turbostratic graphene, characterized by disordered stacking of the graphene layers. The overlapping and curved sheets observed are indicative of turbostratic graphene, where the layers are misaligned compared to crystalline graphite. Some regions exhibit a more disordered structure, revealing the coexistence of amorphous carbon and graphitic domains, a typical feature of biomass-derived carbon materials due to incomplete graphitization. These variations in structure are likely to affect the material’s suitability for specific applications. The high surface area and porosity, visible through the holes, folds, and wrinkled edges in the images, are beneficial for energy storage applications, such as batteries and supercapacitors, as well as gas adsorption, particularly for CO_2_ capture [61,62]. Samples such as the bagasse, cassava pomace, and palm bunches exhibit more distinct lattice fringes, suggesting a relatively higher degree of graphitization. When compared to the carbon black sample, which shows a more uniform, aggregated, and amorphous morphology, the biomass-derived materials present more sheet-like, layered structures, further highlighting their potential in energy storage, catalysis, and adsorption technologies.

### 4.3. Chemical Composition and Functionalization

X-ray photoelectron spectroscopy (XPS) and Near-Edge X-Ray Absorption Fine Structure (NEXAFS) analyses were employed to investigate the chemical composition and atomic structure of the synthesized turbostratic graphene. The XPS revealed the presence of sp^2^-hybridized carbon bonds at 284.5 eV, confirming the formation of graphene-like structures. Additionally, the presence of carbon–oxygen bonds, including hydroxyl (C–O), epoxy (C–O), and carboxyl (O–C=O) groups, indicates partial oxidation during the synthesis process. These oxygenated functional groups are commonly observed in graphene derived from biomass precursors and significantly influence the transformation of the material during the Joule heating process. The transformation mechanism involves the decomposition of these oxygen functionalities under high-temperature conditions, which facilitates graphitization and enhances the conductivity of the material. Oxygen-containing functional groups, although disrupting the sp^2^ bonding network, increase the surface reactivity and contribute to the versatility of the material for various applications, such as sensors, supercapacitors, and energy storage devices. By decomposing during the synthesis, these oxygen functionalities help form a more ordered graphitic structure, which is crucial for optimizing the electronic and mechanical properties of the graphene.

The NEXAFS results further corroborate the presence of sp^2^-hybridized carbon in the synthesized turbostratic graphene. The π* resonance at 284.5 eV, associated with the unoccupied π* states of sp^2^ carbon, confirms the high degree of graphitic ordering. Secondary features observed at higher binding energies suggest the presence of oxygen-containing functional groups, which may originate from the biomass feedstock. These functional groups are beneficial for enhancing the material’s compatibility with various applications where the surface functionalization plays a key role in performance. Thus, the oxygen functionalities introduced during the synthesis process not only influence the structural properties but also enable the fine-tuning of the material’s electronic characteristics, allowing it to be optimized for specific applications.

### 4.4. Influence of Biomass Composition

The elemental composition of the biomass waste used as a precursor has a significant impact on the yield and quality of the synthesized turbostratic graphene. Biomass with higher carbon content, such as palm bunches, produces graphene with better structural ordering and fewer defects. On the other hand, biomass with higher levels of insulating elements like silicon and calcium, such as cassava pomace, impedes the efficient transformation of carbon into graphene. These elements hinder the conductivity of the precursor during the FJH process, requiring a higher energy input to achieve the desired temperature for graphene formation. This suggests that optimizing the biomass composition and carbon content is critical for enhancing the efficiency and scalability of the graphene production process.

### 4.5. Industrial Applications

The successful synthesis of high-quality turbostratic graphene from various biomass waste sources has important implications for sustainable graphene production. Biomass waste is a readily available and renewable source, making it a promising alternative to traditional methods of graphene synthesis that rely on non-renewable resources. The ability to produce graphene with varying degrees of order and functionalization opens up a range of potential applications, including energy storage, catalysis, and environmental remediation. The scalability and cost-effectiveness of the DC-LPFJH process, combined with the use of biomass waste, make it an attractive option for the large-scale production of graphene. One of the primary challenges is achieving uniform heating during the Joule heating process. While the DC-LPFJH technique has proven effective at small scales, ensuring consistent material properties at larger scales remains a challenge. Variations in heating could lead to inconsistencies in the final graphene product, affecting the overall quality and performance of the material. To overcome this limitation, further optimization of the heating process is required, including improved control over the temperature distribution and energy input. Another consideration is the cost of biomass preprocessing and the energy required to maintain the necessary reaction conditions. Biomass waste with high levels of carbon content is preferred for achieving high-quality graphene; however, the presence of impurities or insulating elements in some biomass waste may hinder the efficient transformation into graphene. This could result in higher energy requirements, impacting both the scalability and economic viability of the process. Therefore, optimizing the biomass composition, pre-treatment, and reaction conditions will be essential for reducing the energy consumption and improving the cost-effectiveness of biomass-derived graphene production.

Selecting appropriate biomass wastes and optimizing the process parameters highlights the versatility and potential of the DC-LPFJH technique. Continued research into the process optimization, particularly regarding the energy efficiency and achieving uniform heating, will be essential for enhancing both the scalability and economic viability of the method. By addressing these limitations, biomass-derived graphene has the potential to become a viable, sustainable material for a wide range of industrial applications, from energy storage and catalysis to environmental remediation.

### 4.6. Comparison with Previous Studies

Table 3 compares the turbostratic graphene production of several selected Flash Joule Heating techniques. It should be noted that the information and values presented here were obtained and calculated from data provided in the references.

The most notable difference is the use of biomass waste as the carbon precursor in this work, as opposed to conventional sources such as coal, petroleum coke, or plastics. This sustainable choice not only reduces waste, but also avoids the use of solvents, making the process more environmentally friendly. The energy requirement for the biomass-based production process in this study (10 kJ/g) is significantly lower than that of other processes, such as those using mixed plastic waste (23 kJ/g), and comparable to those using coal (7.2 kJ/g), highlighting the efficiency of the DC-LPGH method employed here. However, the structural analysis via Raman spectroscopy and TEM analysis reveals that our process tends to produce lower I_2D_/I_G_ ratios, likely due to the longer heating duration involved in the DC-LPGH method. This results in a graphene product with unique characteristics in terms of morphology, including multilayer graphene and a relatively higher degree of disorder compared to other methods.

## 5. Conclusions

This study demonstrates the successful synthesis of high-quality turbostratic graphene from biomass waste using the DC-LPFJH technique. Raman spectroscopy, TEM, XPS, and NEXAFS analyses provide strong confirmation of the material’s graphene-like nature, revealing partial oxidation, varying degrees of graphitization, and the presence of oxygen-containing functional groups. The distinct 2D band observed in the Raman spectra and the TEM images confirm the formation of thin, layered graphene structures, while the XPS and NEXAFS results highlight the influence of the functional groups on the material’s hydrophilicity, reactivity, and potential for various applications. The elemental composition of the biomass significantly affects both the yield and the quality of the resulting graphene. High-carbon-content biomass sources lead to better-ordered graphene with fewer defects, whereas feedstocks with higher oxygen content result in more functionalization, enhancing the material’s surface reactivity. The DC-LPFJH process plays a pivotal role in driving the structural transformation, with the oxygen functionalities facilitating the graphitization process, which is essential for improving the electrical conductivity. Although the method shows promise for scalable graphene production, challenges remain in ensuring uniform heating and controlling the material properties across larger quantities. Further optimization of the Long Pulse Joule Heating technique is necessary to improve the precursor conductivity and address the environmental concerns related to the process.

Future research should focus on enhancing the electronic and chemical properties of biomass-derived graphene through heteroatom doping, assessing its mechanical properties for use in composite materials, and evaluating its electrochemical performance in energy storage applications. The potential for environmental applications, such as CO_2_ capture and pollutant adsorption, along with its catalytic and sensing capabilities, should also be explored to broaden its applicability. Addressing these research directions will establish biomass-derived turbostratic graphene as a sustainable, high-performance material with wide-ranging applications in advanced technological fields.

## Figures and Tables

**Table 2:** XRF analysis of elemental composition of biomass waste, before and after carbonization process.

Elements	Types of Biomass Waste													
	Bagasse		Cassava Pomace		Sugarcane Leaves		Straw		Palm Bunches		Corncobs		Corn Stalks	
	Before	After	Before	After	Before	After	Before	After	Before	After	Before	After	Before	After
C	47.82	79.51	44.25	40.04	47.26	53.47	36.42	48.37	50.02	80.29	47.32	72.25	45.45	70.61
O	50.4	11.55	50.36	21.94	48.04	22.94	53.55	31.08	46.66	9.29	50.81	24.11	46.85	13.57
Na			0.02					0.06		0.05				0.02
Mg	0.04	0.26	0.44	1.26	0.2	0.79	0.14	0.32	0.16	0.28	0.10	0.23	0.17	0.33
Al	0.13	0.50	0.13	0.56	0.03	0.08	0.14	0.27	0.04	0.04	0.01	0.01	0.03	0.04
Si	1.22	5.70	0.56	6.18	2.83	13.18	7.16	15.19	0.68	0.83	0.74	1.34	1.69	2.69
P	0.03	0.20	0.16	1.07	0.05	0.28	0.09	0.25	0.10	0.17	0.09	0.21	0.16	0.30
S	0.04	0.13	0.09	0.37	0.14	0.83	0.07	0.06	0.10	0.08	0.04	0.04	0.10	0.04
Cl	0.02		0.06	0.25	0.07	0.04	0.22	0.13	0.30	0.97	0.36	0.09	1.17	1.86
K	0.13	0.79	1.24	4.83	0.61	2.97	1.57	2.68	1.601	3.40	0.45	1.48	1.86	3.33
Ca	0.09	0.72	2.58	22.24	0.72	5.13	0.45	1.14	0.19	0.27	0.05	0.17	0.36	0.57
Ti		0.07		0.08					0.02	0.02		<0.01		
Cr		0.01					0.06	0.16	<0.01	<0.01			0.01	0.02
Mn	0.01	0.03	0.01	0.09	0.02	0.12	0.09	0.24	0.09	0.14	0.01	0.04	0.01	0.03
Fe	0.06	0.52	0.1	1.01	0.02	0.15						<0.01		
Ni		0.01					<0.01	0.01		<0.01	<0.01	0.02	<0.01	<0.01
Zn			<0.01	0.02						<0.01			<0.01	<0.01
Rb				0.01					<0.01	<0.01				<0.01
Sr			<0.01	0.03	<0.01	0.01	<0.01	<0.01		<0.01				<0.01

**Table 3:** Comparison of turbostratic graphene production methods from various carbon precursors.

Feature	[25]	[27]	[29]	This Work
Carbon Precursor	Various carbon sources (coal, petroleum coke, biochar, plastics, etc.)	Mixed plastic waste (HDPE, PET, PVC, etc.)	Anthracite coal	Biomass
Process	Flash Joule Heating	Alternating Current + Direct Current Flash Joule Heating (AC-DC FJH)	Flash Joule Heating	Direct Current Long Pulse Joule Heating (DC-LPGH)
Energy Requirement	~7.2 kJ/g	~23 kJ/g	Not explicitly stated	18 kJ/g
Graphene Type	Turbostratic graphene	Turbostratic graphene	Turbostratic graphene	Turbostratic graphene
Raman Spectroscopy	I_2D_/I_G_ up to 17 (high-quality graphene)	I_2D_/I_G_ up to 6 (after DC-FJH treatment)	Confirmed turbostratic structure, peak shift at 26.0°	Typically lower I_2D_/I_G_ due to longer heating duration
TEM Analysis	Graphene layers show Moiré patterns	Layer structure confirmed; fewer defects after DC-FJH	Larger interlayer spacing than typical graphite	Multilayer graphene
Environmental Impact	Sustainable, no solvents required	Upcycling of plastic waste, prevents pollution	Efficient use of coal, reduces waste	Sustainable, no solvents required, reduces biomass waste
Potential Applications	Composite materials	Composite materials	Energy storage, catalysis, carbon-based applications	Composite materials

## Data Availability

The original contributions presented in this study are included in the article. Further inquiries can be directed to the corresponding author.
